# Vitamin D and Respiratory Tract Infections: A Systematic Review and Meta-Analysis of Randomized Controlled Trials

**DOI:** 10.1371/journal.pone.0065835

**Published:** 2013-06-19

**Authors:** Peter Bergman, Åsa U. Lindh, Linda Björkhem-Bergman, Jonatan D. Lindh

**Affiliations:** 1 Department of Laboratory Medicine, Division of Clinical Microbiology, Karolinska Institutet, Stockholm, Sweden; 2 Department of Medicine, Center for Infectious Medicine (CIM), Karolinska Institutet, Stockholm, Sweden; 3 Northern Stockholm Psychiatry, St. Göran Hospital, Stockholm, Sweden; 4 Department of Laboratory Medicine, Division of Clinical Pharmacology, Karolinska Institutet, Stockholm, Sweden; Copenhagen University Hospital Gentofte, Denmark

## Abstract

**Background:**

Low levels of 25-OH vitamin D are associated with respiratory tract infection (RTI). However, results from randomized controlled trials are inconclusive. Therefore, we performed a systematic review and meta-analysis to assess the preventive effect of vitamin D supplementation on RTI.

**Methods:**

Randomized, controlled trials of vitamin D for prevention of RTI were used for the analysis. The risks of within-trial and publication bias were assessed. Odds ratios of RTI were pooled using a random-effects model. Heterogeneity was assessed using Cochran's Q and I^2^. Meta-regressions and subgroup analyses were used to assess the influence of various factors on trial outcome. The pre-defined review protocol was registered at the PROSPERO international prospective register of systematic reviews, registration number CRD42013003530.

**Findings:**

Of 1137 citations retrieved, 11 placebo-controlled studies of 5660 patients were included in the meta-analysis. Overall, vitamin D showed a protective effect against RTI (OR, 0.64; 95% CI, 0.49 to 0.84). There was significant heterogeneity among studies (Cohran's Q p<0.0001, I^2^ = 72%). The protective effect was larger in studies using once-daily dosing compared to bolus doses (OR = 0.51 vs OR = 0.86, p = 0.01). There was some evidence that results may have been influenced by publication bias.

**Interpretation:**

Results indicate that vitamin D has a protective effect against RTI, and dosing once-daily seems most effective. Due to heterogeneity of included studies and possible publication bias in the field, these results should be interpreted with caution.

## Introduction

Respiratory tract infections (RTIs) are common worldwide and are responsible for significant morbidity and mortality. According to a recent report, 2.8 million deaths were caused by RTI during 2010 [1]. The most common causal agents are the bacterium *Streptococcus pneumoniae* and influenza-virus. Vaccination against these microbes is available in certain areas of the world. However, this preventive measure may not be completely protective due to non-responders and microbial vaccine escape mechanisms. Treatment options include symptomatic treatment, antibiotics and antivirals, although the emerging resistance may limit this possibility in the future. Thus, additional ways to prevent or ameliorate RTIs are needed and modulation of the host immune response could provide such an innovative approach.

Recent evidence suggests that vitamin D influences several immune pathways, with the net effect of boosting mucosal defenses while simultaneously dampening excessive inflammation [2]. For example, vitamin D induces the gene encoding the antimicrobial peptide LL-37 [3]. This peptide has potent bactericidal capacity against a number of important bacteria and viruses, including *M. tuberculosis* and influenza-virus [4], [5]. In fact, human macrophages rely upon the vitamin D/LL-37-axis to kill mycobacteria, an effect that is abrogated if the LL-37 gene is silenced with RNA-interference [6], [7].

In humans, the main source of vitamin D is UVB-mediated synthesis in the skin. Certain food, such as oily fish and dairy products, contains vitamin D, but it is difficult to achieve sufficient intake by the diet alone. The activation of vitamin D involves two hydroxylation steps, one in the liver and one in the kidney. Notably, the final activation of vitamin D, via 1-alpha hydroxylase (CYP27B1), also occurs in extra-renal tissues, including epithelial and immune cells [8]. In the respiratory tract, CYP27B1 is expressed in bronchial epithelial cells and induced by inflammatory stimuli [9]. Thus, the vitamin D/antimicrobial peptide-circuit may be activated locally upon infection, which further suggests a role for vitamin D in host defense.

Additional evidence supporting a role for vitamin D in respiratory tract infections is provided by observational reports showing an association between low 25-OH vitamin D (25(OH)D) levels and increased risk of infection. A large cross-sectional trial (n = 18883) showed that the risk of RTI increased with lower 25(OH)D levels and that the effect was even stronger in individuals with chronic obstructive pulmonary disease (COPD) or asthma [10]. In addition, many case-control studies report clear associations between low 25(OH)D levels and increased risk of RTI (reviewed in [11]). Since observational studies can be questioned due to hidden bias effects, randomized controlled interventional studies are needed to infer causality.

However, published randomized controlled trials (RCTs) addressing the hypothesis that vitamin D could prevent RTI are not conclusive. A systematic review and meta-analysis was recently published and found a significant effect of vitamin D supplementation against RTI in children but not in adults [12]. This study only included 5 clinical trials in the analysis, which could have affected the result. Another systematic review (without meta-analysis) have included both observational and interventional trials and discussed potential explanations for the diverging results in previous trials [11]. For example, results may have been influenced by the choice between daily or bolus doses, by baseline 25(OH)D levels, and by RTI being a primary or secondary endpoint. However, the quantitative impact of these factors has not been analyzed. Moreover, many of the published RCTs are small, and the expected random variability among trials has not been discussed, nor has the potential influence of publication bias. To address these questions, we performed a systematic review and a meta-analysis of published RCTs, including data from a recently published RCT from our own group.

## Methods

### Eligibility criteria

Eligible for inclusion were randomized comparisons of vitamin D and placebo or no treatment, reporting incident respiratory tract infection as a primary or secondary outcome. Studies addressing tuberculosis or fungal infections were excluded since these clinical entities were considered to be biologically and medically distinct from RTIs, but otherwise there were no restrictions regarding type of infectious agent. There was no distinction made between “upper” and “lower” RTI and thus the description “RTI” designates both these entities. Studies reporting composite endpoints deemed to mainly reflect infectious episodes were also considered for inclusion. Eligible outcomes included relative measures of infection risk (relative risk or odds ratio) or absolute numbers of patients experiencing at least one episode of RTI. If these measures were not available, studies reporting number of RTI episodes or days with RTI per patient were also considered available for inclusion, as were studies reporting indirect measures of incident RTI (e.g. cumulative RTI symptom scores or RTI-associated absence from work or school). There were no limitations with regard to patient characteristics, vitamin D dose, treatment duration, year of publication or language of publication.

### Search strategy and data extraction

Information sources included Medline, Embase, Web of science, the Cochrane central register of controlled trials, congress abstracts and review article reference lists (up to April 15, 2013). In Medline, MeSH-indexed publications were searched with the following query: “Vitamin D”[MeSH] AND (“Respiratory Tract Infections”[MeSH] OR “Infection”[MeSH]). For publications which had not yet been subjected to MeSH-indexing the following query was used: (“vitamin D” OR “ergocalciferol” OR “cholecalciferol” OR “alfacalcidol”) AND infection AND (publisher[sb] OR in process[sb]). Embase, was searched using the query: ‘vitamin D’/exp AND ‘respiratory tract infection’/exp AND ‘clinical trial’/exp AND [embase]/lim; Web of science: (“vitamin D” OR ergocalciferol OR cholecalciferol OR alfacalcidol) AND infection AND randomized; Chochrane central register of controlled trials: ([mh “Respiratory Tract Infections”] OR [mh Infection]) AND [mh “Vitamin D”] (restricted to trials).

Titles and abstracts of records identified in the primary search were screened by a single investigator and all articles deemed potentially eligible for inclusion were retrieved in full-text format. Extraction of necessary data (including e.g. authors, publication year and journal, population characteristics, vitamin D doses and routes of administration, trial duration and outcome measures) was performed independently by two investigators and any discrepancies were resolved by consensus. A full list of extracted data items are presented in table S1. The pre-defined review protocol was registered at the PROSPERO international prospective register of systematic reviews (http://www.crd.york.ac.uk/PROSPERO, registration number CRD42013003530). The protocol for this trial and supporting CONSORT checklist are available as supporting information; see Checklist S1 and Protocol S1.

### Assessment of methodological quality

The methodological quality and risk of bias in individual trials were assessed by means of the Cochrane Collaboration's tool for assessing risk of bias in randomized trials [13]. The assessment tool covers a range of bias mechanisms, including selection, performance, detection, attrition, and reporting bias. A summary assessment was made, where studies with high risk of bias in one or more of these items were deemed to be at a high overall risk of bias.

### Statistical analyses

The principal summary measure was the odds ratio of RTI in vitamin D-treated individuals as compared to recipients of placebo. Continuous indices of RTI burden were transformed from standardized mean differences to odds ratios [14] using the Meta-Analysis Effect Size Calculator by DB Wilson [15]. Odds ratio estimates from eligible studies were summarized in a random-effects (DerSimonian-Laird) meta-analysis weighing each trial according to the inverse standard error of its log-transformed OR estimate. Heterogeneity among studies was assessed by means of the Cochran's Q test (at a significance level of 0.10) and by calculating I^2^ (the proportion of variability across studies attributable to heterogeneity rather than chance).

Since the meta-analysis was based on relative measures of effect, it was not possible to calculate an absolute treatment effect or number needed to treat (the number of subjects one would have to treat for a specified length of time in order to avoid a single episode of RTI). However, some of the included studies did present the absolute risk of RTI in the control group, and by combining these risk estimates with the overall OR from the meta-analysis it was possible to calculate a range of NNTs as a rough estimate of the NNTs in the populations under study.

When published data is still sparse and repeated cumulative meta-analyses are performed as new data becomes available, there is a substantial risk of spurious false-positive findings when assessing statistical significance at the 0.05 level. As a general principle, p-values just below 0.05 should only be considered statistically significant if the amount of information available equals that which would be required in a single trial sufficiently powered to detect a clinically relevant effect at a significance level of 0.05. If the amount of information available for meta-analysis is still below this *required information size*, an alpha-spending function can be used to calculate alternative significance thresholds capable of maintain the risk of false positives at a level of 5% [16]. To investigate whether available evidence was sufficient to analyze data at a significance level of 0.05 with a power of 80%, we calculated required information sizes (number of participants) based on a range of assumptions regarding risk of RTI in the control group (25–75%), relative risk reduction in the vitamin D group (25–50%), and level of heterogeneity (0–75%).

Publication bias was detected by visual inspection of funnel plots and asymmetries were assessed further with the Begg-Mazumdar and Egger tests. To identify randomized controlled studies whose results had remained unpublished (potentially due to selective non-publishing of negative or inconclusive results), the NIH clinical trial registry (www.clinicaltrials.gov) was searched using the following query: “infection AND vitamin D”. Identified trial registrations were manually searched for studies addressing prevention of RTI and among those, trials lacking published results despite a scheduled completion more than one year ago were recorded.

The potential impact of various patient and trial-level parameters on the trial outcome was investigated by means of pre-specified, univariable random effects meta-regressions. In these regressions, log-transformed odds ratios were regressed on the following variables: pre-treatment 25(OH)D levels in serum, latitude of the trial site, vitamin D dose, administration once daily (vs bolus doses), RTI as primary outcome (vs secondary), and gender distribution and mean age of the trial participants. In the meta-regressions, studies were weighed according to inverse standard error. In addition, the influence of binary predictors was investigated in subgroup analyses where the overall meta-analysis was repeated separately for each subgroup of trials, as well as for trials with low and high risk of bias. Finally, the influence of single studies was investigated in an influence analysis where pooled estimates were recalculated after omitting one trial at a time and the main analysis was repeated after inclusion of initially excluded studies which failed to fulfill all inclusion criteria but presented evaluable RTI data.

Required information size was calculated using the Trial Sequencial Analysis (TSA) software (http://www.ctu.dk/tsa/) [16]. All other statistical analyses were performed using R 2.15.0 (R Development Core Team (2012). R: A language and environment for statistical computing. R Foundation for Statistical Computing, Vienna, Austria. ISBN 3-900051-07-0, URL http://www.R-project.org/.), packages Epi, meta, metaphor, and rmeta.

## Results

### Included studies

**Figure 1:**
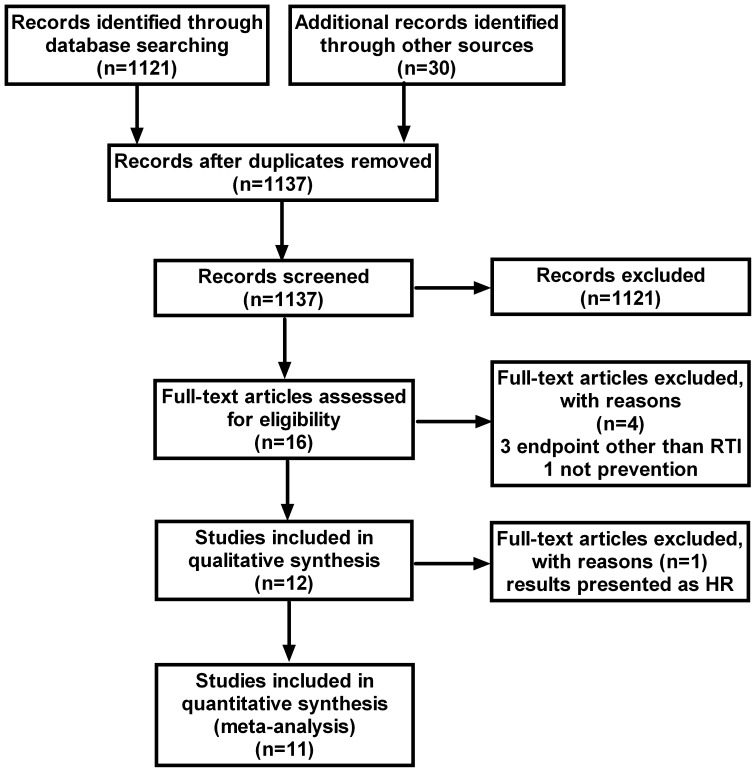
Flowchart for selection of eligible studies.

The literature search identified a total of 1137 studies (figure 1). Sixteen of these were retrieved in full-text [17]–[32] and 11 were included in the final analysis [17], [19], [21], [23], [25]–[32]. The characteristics of the included studies are summarized in table 1. Three studies [18], [20], 24 were excluded since they reported infections in general, without specifying RTIs separately. In addition, one of these studies compared two different doses of vitamin D and consequently lacked a placebo group [20]. A fourth trial was not included since it did not study the preventive effect of vitamin D, but rather the therapeutic effect in patients with manifest pneumonia [22]. A fifth trial was eligible for inclusion in the review, but was excluded from the meta-analysis, since the outcome was presented as hazard ratio incompatible with the outcome measures of the remaining 11 studies [26]. In the latter trial, the hypothesis was that high bolus doses of 100,000 IU vitamin D_3_ every 4 weeks could prevent exacerbations in chronic obstructive pulmonary disease (COPD). The result was negative with regards to the primary outcome, which was time to first exacerbation (hazard ratio 1.1, 95% CI 0.82–1.56). However, a post-hoc analysis revealed a significant effect (rate ratio 0.57, 95% CI 0.33–0.98) in the 30 participants with 25(OH)D levels below 25 nmol/L.

**Table 1:** Characteristics of included studies.

1st author	Year	Country	Latitude	Administration route	Average daily dose (IU)	Follow-up time	n (vit D)	n (placebo)	n (total)	Primary endpoint	Healthy participants	Bacterial infection	Daily administration	Men	Women	Age (years)	25OH Vit D conc baseline (vit D) (nmol/L)	25OH Vit D conc follow-up (vit D) (nmol/L)	25OHVit D conc baseline (placebo) (nmol/L)	25OHVit D conc follow-up (placebo) (nmol/L)
**Aloia ** [**17**]	2007	USA	42 N	oral	800/2000¤	3 y	104	104	208	No	Yes	No	Yes	0	1	60·6	46·9	86·9	43	43
**Bergman ** [**19**]	2012	Sweden	59 N	oral	4000	12 mo	62	62	124	Yes	No	No	Yes	0·27	0·73	53·1	51·5	117·4	46·9	44
**Camargo ** [**21**]	2012	Mongolia	48 N	oral	300	7 w	141	103	244	No	Yes	No	Yes	0·52	0·48	9·97	17·5	47·3	17	18
**Jorde ** [**23**]	2012	Norway†	#	oral	3344	12 w	289	280	569	No	Yes	No		0·57	0·43	63	ND	ND	ND	ND
**Laaksi ** [**25**]	2010	Finland	61 N	oral	400	6 mo	80	84	164	Yes	Yes	No	Yes	1	0	*	78·7	72	74·4	51
**Lehouck ** [**26**]	2012	The Netherlands	50 N	oral	3333	12 mo	91	91	182	Yes	No	Yes	No (A)	0·8	0·2	68	50	130	50	55
**Li-Ng ** [**27**]	2009	USA	41 N	oral	2000	3 mo	78	70	148	Yes	Yes	No	Yes	0·2	0·8	58·7	64·3	88·5	63	60·9
**Majak ** [**28**]	2011	Poland	51 N	oral	500	6 mo	24	24	48	Yes	No	No	Yes	0·67	0·33	11·5	90	94	88	80
**Manaseki-Holland ** [**30**]	2010	Afghanistan	33 N	oral	NA	3 mo	224	229	453	Yes	No	Yes	No (B)	0·56	0·44	1·2	ND	ND	ND	ND
**Manaseki-Holland ** [**29**]	2012	Afghanistan	33 N	oral	1296	18 mo	1524	1522	3046	Yes	Yes	Yes	No (C)	0·52	0·48	0·8	ND	‡	ND	§
**Murdoch ** [**31**]	2012	New Zealand	43 S	oral	3653	18 mo	161	161	322	Yes	Yes	No	No (D)	0·25	0·75	47·5	72·5	122·5	70	55
**Urashima ** [**32**]	2010	Japan	40 N	oral	1200	4 mo	167	167	334	Yes	Yes	No	Yes	0·56	0·44	10·2	ND	ND	ND	ND

* “young Finnish men”, “homogenous with regards to age”;

† Norway, Denmark, Belgium, US, Austria, Scotland; #various latitudes;

‡ significantly higher than placebo;

§ significantly lower than vitamin D group, ¤800 IU/year for 2 years and 2000 IU/year during the 3rd year.

Administration interval: A, 100,000 IU/4 weeks; B, 100,000 once; C, 100,000 IU/3 months; D, 200,000 IU initially, 200,000 after 1 month and thereafter 100,000/month. The study by Lehouck et al (24) was not included in the meta-analysis, see Materials and Methods for details.

A total of 5660 patients were included in the 11 studies (50% men and 50% women), with an average age of 16 years. The average vitamin dose was 1600 IU/day and the dose interval varied between 24 hours and 3 months. One trial used a single dose of 100,000 IU [30]. All studies were placebo-controlled and used orally administered cholecalciferol (vitamin D3).

The included studies were generally of high methodological quality, although the risk of attrition bias due to incomplete outcome data was unclear in several studies (table S2). Only two trials were judged to be at a high risk of bias [17], [23].

Based on a range of assumptions regarding baseline risk, treatment effect and heterogeneity, the calculated number of participants required to provide firm evidence of clinically relevant treatment effect ranged from <200 to 5496 patients. Since the actual number of patients in the meta-analysis (5600) exceeded these numbers, it was concluded that an unadjusted significance threshold level of 0.05 (two-sided test) was justifiable.

### Synthesis of results

**Figure 2:**
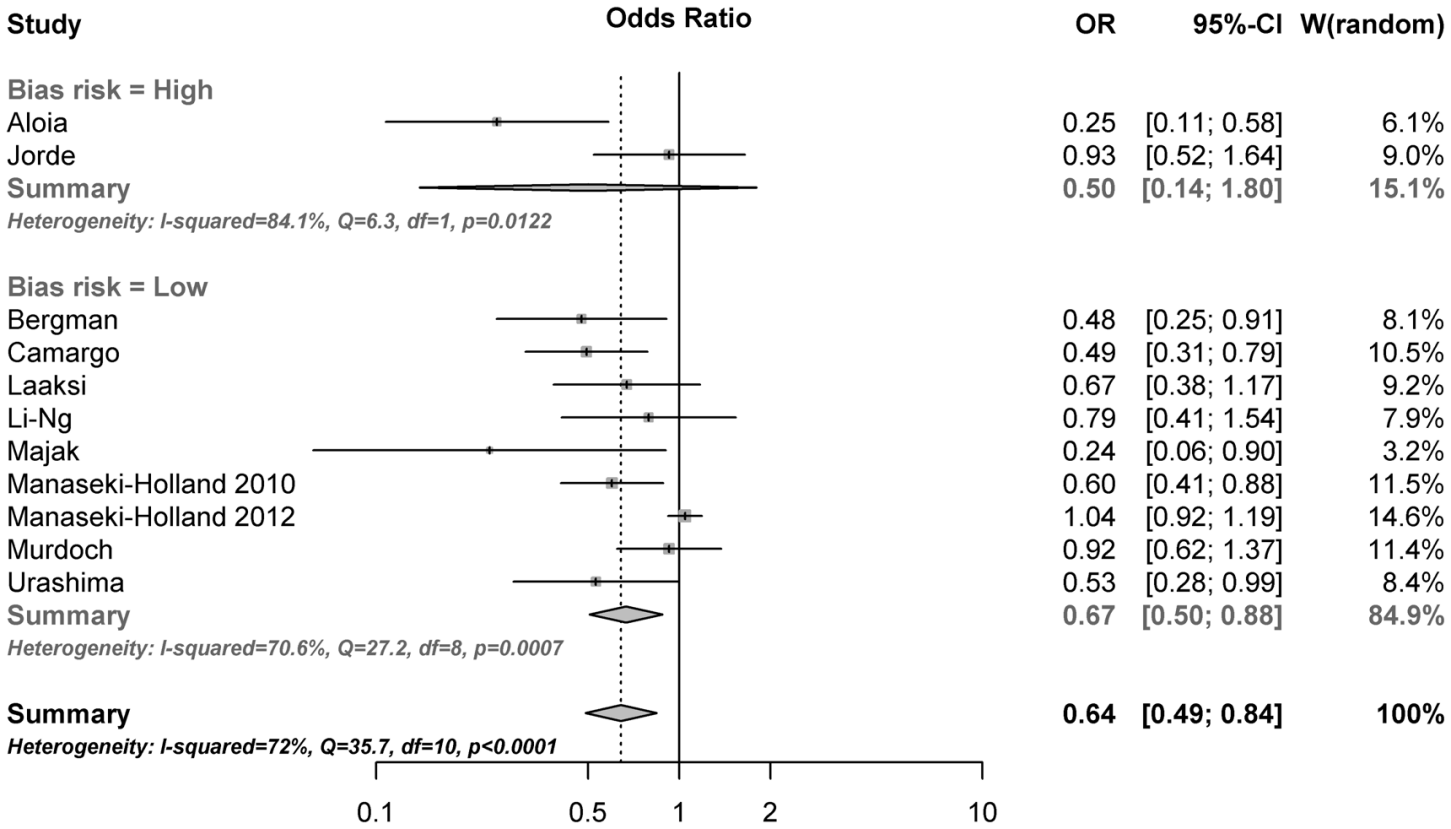
Efficacy of vitamin D for prevention of respiratory tract infections. Error bars indicate 95% confidence intervals.

The results of the overall meta-analysis are presented in figure 2. The summarized results of the 11 included randomized trials indicates that substitution with vitamin D significantly reduces the risk of respiratory tract infections (OR, 0.64; 95% CI, 0.49 to 0.84; p = 0.0014). There was evidence of a significant heterogeneity among studies (Cochran Q = 35.7; p<0.0001, I^2^ = 72%), confirming the need for a random effect model. The observed effect of vitamin D was larger in studies with a high risk of bias (OR, 0.50; 95% CI, 0.14 to 1.80), compared to studies with a low risk of bias (OR, 0.67; 95% CI, 0.50 to 0.88), but this difference was not statistically significant (p = 0.67 for subgroup difference). According to the influence analysis (figure S1), each trial had a modest influence on the overall results, and after exclusion of single studies the estimated OR remained within the range 0.61 to 0.69.

### Risk of bias across studies

A funnel plot of included studies is presented in figure S2. A large treatment effect in the two trials with the lowest precision and the lack of effect in the trial with highest precision could be indicative of publication bias. Indeed, the Egger test for funnel plot asymmetries was highly significant (p<0.001), but the non-parametric Begg-Mazumdar test was not (p = 0.14). By searching the clinical trial registry www.clinicaltrials.gov for studies on “vitamin D AND infection”, we identified 181 studies, 25 of which included clinical conditions related to respiratory tract infections, including influenza, asthma or chronic obstructive pulmonary disease as a primary endpoint. The majority was either “completed” or “ongoing”, 3 interventional studies (NCT01158560, NCT01215682, NCT01549938) and 1 observational trial (NCT01486160) had been completed during 2012 and results had not yet been published (Jan, 2013). We did not find any completed trial older than 2011 with unpublished results. Inclusion of three initially excluded trials with evaluable data on RTIs [18], [20], [24] had a modest effect on the overall results (OR, 0.72; 95% CI, 0.60 to 0.87).

### Additional analyses

**Figure 3:**
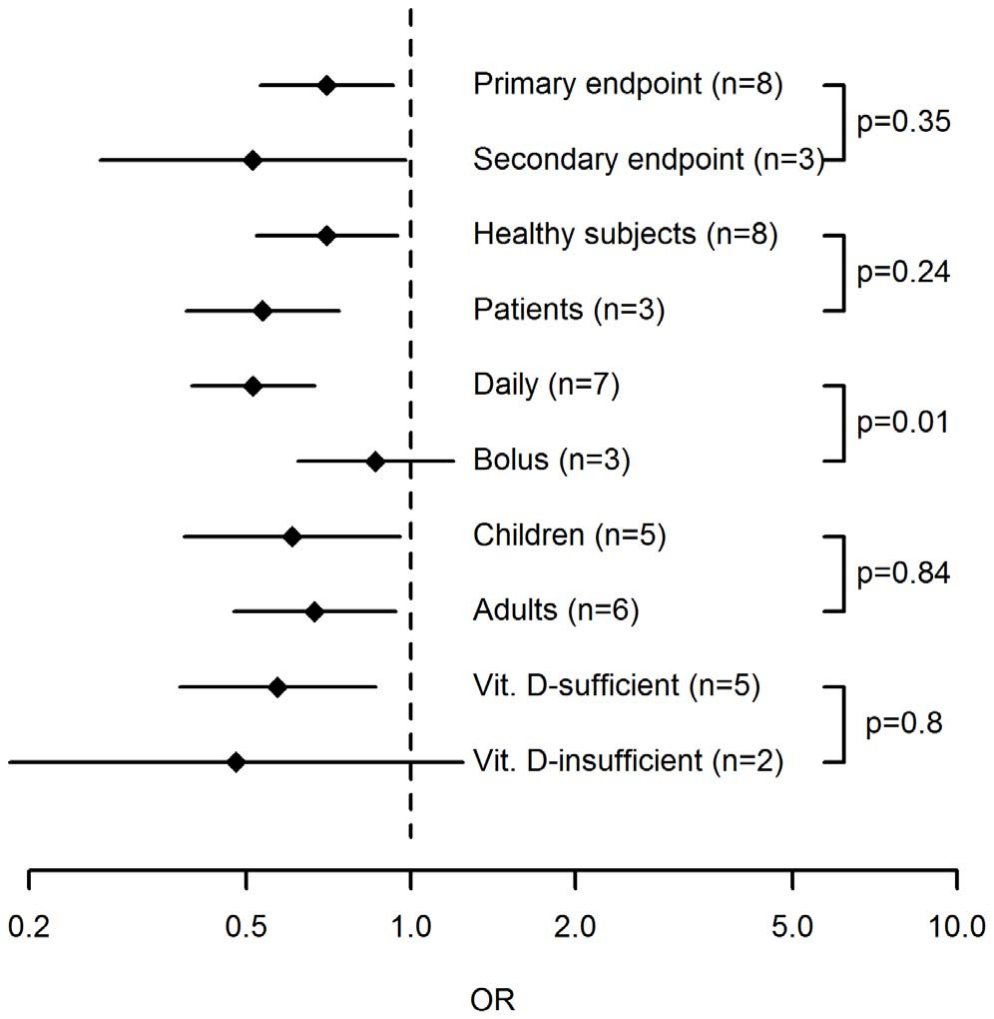
Subgroup analyses. Error bars indicate 95% confidence intervals of OR in subgropus of randomized trials. Subgroups were based on RTI being a primary or secondary endpont, trial participants being patients or healthy individuals, children or adults, and vitamin D-sufficient or insufficient, and vitamin D being adminstered daily or as bolus doses. Numbers indicate number of trials in each subgroup and p-values refer to between-group differences in random effects meta-regressions performed separately for each pair of subgroups.

**Figure 4:**
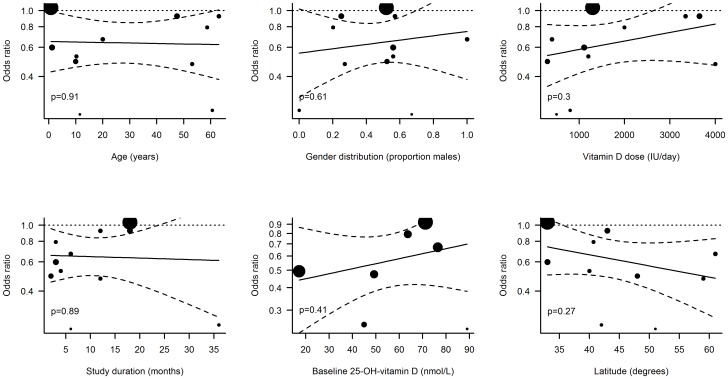
Random effects meta-regressions. Dotted lines indicate 95% confidence intervals of regression lines (solid lines). Sizes of dots are proportional to the weight of each trial in the regression model.

In the meta-regressions performed, the administration interval turned out to be a significant predictor of vitamin D effectiveness in preventing RTI (figure 3). In studies where vitamin D was administered daily, the treatment was associated with a significant reduction in RTIs (OR, 0.51; 95% CI, 0.39 to 0.67) while vitamin D had no effect when administered in large bolus doses once per month or less frequently (OR, 0.86; 95% CI, 0.62 to 1.20). The effect of administration interval was statistically significant in a random effects regression model (p = 0.01). None of the other trial-level parameters investigated were significant predictors of vitamin D effectiveness, including if the endpoint was primary or secondary (p = 0.35), if healthy individuals or patients were studied (p = 0.24) as well as age (p = 0.91 for age, p = 0.84 for children vs adults), gender (p = 0.61), dose (p = 0.3), trial duration (p = 0.89), baseline 25(OH)D levels (p = 0.43 for concentration, p = 0.80 for <75 vs ≥75 nmol/L) or latitude (p = 0.27) (Figure 4). We further compiled data on reported adverse events in the included trials (table S3). Only four of these reported any adverse events (AE) and 3 trials defined severe adverse events (SAE) as a separate entity. None of the reported AEs or SAEs was considered to be related to the study drug.

In studies presenting absolute numbers of events per study group, the absolute risk of RTI ranged from 9% [23] to 58% [30] over a three-month period. By combining these boundaries with the OR from the overall meta-analysis, NNTs ranging from 9 to 33 were calculated.

## Discussion

### Summary of evidence

Our meta-analysis of randomized controlled trials indicates a protective effect of vitamin D supplementation against respiratory tract infections with a combined odds ratio of 0.64 (95% CI 0.49–0.84). Although the overall results were in favor of a vitamin D effect, there was significant heterogeneity among studies. To address this heterogeneity, we performed a number of meta-regressions and subgroup analyses investigating the influence of trial characteristics on the observed vitamin D effect. According to these analyses, the dosing interval appeared to be a key factor and studies using daily doses of vitamin D showed significantly better therapeutic effect than studies where participants were given large bolus doses of vitamin D at long intervals (1–3 months). As pointed out by Heaney [33], Martineau [34] and Hollis [35] there may be a biological explanation to a smaller effect when using a bolus schedule. At high doses, vitamin D is in fact immunosuppressive, a phenomenon that is utilized in trials on vitamin D and inflammatory disorders, such as multiple sclerosis. A trial where 10,000 IU/day were given (mean levels of 25(OH)D were 179+/−76 nmol/L) clearly showed that proliferative responses of peripheral blood monocytes (PBMC) were suppressed [36]. Further, vitamin D suppressed inflammation, both *in vitro* [37] and *in vivo* [38] but the clinical consequences remain to be determined. It could, however, be speculated that very large doses of vitamin D could have adverse effects on immunity. Notably, the Manaseki-Holland trial from 2012 using large bolus doses of 100,000 IU/3 months, reported that the intervention group had a slightly higher risk of secondary pneumonia [29]. In the trial by Lehouck et al – where 100,000 IU/4 weeks were given – the placebo-group produced significantly *more* positive sputum cultures at baseline than the vitamin D group. This difference was also evident after 4 months but disappeared after 8 and 12 months of vitamin D supplementation, indicating a lack of the spontaneous improvement that the placebo-group experienced ([26], Appendix, table 4). Previously, a cross-sectional trial from Greenland showed that both low (<75 nmol/L) and high serum concentrations (>140 nmol/L) were associated with an increased risk of tuberculosis [39]. Similarly, molecular studies suggest the presence of feedback systems effectively blocking the activation of vitamin D at several levels when large supraphysiological doses are given [40]–[42]. Thus, mechanistic evidence supports administration of vitamin D once daily, unless immunosuppressive effects are wanted. Not only do our results support this notion, but they also provide a quantitative estimate of the effect; i.e. studies using a daily dosing regimen show a 3.5 times larger reduction in the odds of RTI than those using a bolus schedule (OR 0.51 vs 0.86). This could explain why many of the studies using bolus doses have provided null effect and is also important information when designing future interventional studies. However, a bolus scheme could be preferred when compliance is expected to be poor. For example, dosing schemes once a week may be a good compromise to improve effect compared to bolus doses while still facilitating compliance. In fact, large doses of vitamin D (33,000–150,000 IU) ranging from every month to every 4 months have been shown to be efficient in clinical studies of fractures [43] and muscle strength [44]. Thus, even though our data suggest that a daily dosing schedule could be better with regards to endpoints related to infections; more studies addressing this particular issue are warranted.

We also investigated whether age, baseline 25(OH)D levels or disease conditions of the trial population as well as the latitude of the trial site affected the outcome. Although participants represented a large age-span (6 months-75 years), our data do not support any impact of age on the outcome measure. Previous studies have suggested that only those individuals with a low 25(OH)D level may benefit from supplementation [26] and a recent trial in mainly 25(OH)D-replete participants showed no effect against RTI [31]. We could not confirm this association between baseline 25(OH)D levels and outcome of supplementation, but the negative finding should be interpreted with caution due to the limited number of studies analyzed. Lastly, studies including patients did not show a better effect than those including healthy individuals; nor did we find a connection between distance from the equator and effect of vitamin D, which is in line with the findings from a previous trial [45].

In addition to the vitamin D dosage interval, other elements of the trial design, e.g. whether the RTI outcome was a primary or secondary endpoint, the trial duration and the vitamin D dose, were assessed. None of these factors had a significant modulating effect on the effect of vitamin D supplementation. When interpreting the outcome of the meta-regressions, one should bear in mind that the use of aggregated trial-level data provides less statistical power compared to individual-level analyses. Failure to demonstrate a significant association should therefore not be interpreted as evidence against an effect.

As evident from the funnel plot, the smallest effect was observed in the trial with highest precision [29] and the two least precise effect estimates[17], [28] indicated the largest effect of vitamin D supplementation. This tendency towards increasing effect estimates with decreasing precision could be indicative of publication bias, with selective publishing of favorable results. According to the non-parametric Begg-Mazumdar test, this asymmetry was not statistically significant, while the Egger test indicated highly significant asymmetry. However, the pronounced effect in the latter analysis was almost entirely attributable to the influence by a small number of outlier studies, and in this situation results from linear regression models such as the Egger test are known to be unreliable. One of these influential studies was the large Manaseki-Holland trial from 2012 including 3046 children and with showing a null result [29]. The design of this trial has been thoroughly analyzed by Martineau [34] and several reasons for the null effect have been proposed. These include the use of a bolus schedule, the fact that the participants were infants below 6 months of age (with an immature immunity) and the possibility of nutritional deficits other than vitamin D [29]. One of the two studies indicating the largest effect was assessed as being at high risk of bias [17] and the other trial by Majak et al. [28] had a different design where 48 asthma patients were given 500 IU vitamin D or placebo/day for 6 months and the primary endpoint was “exacerbation of asthma”. Thus, the heterogeneity in design between the studies makes it difficult to evaluate to what extent the association between precision and effect size estimate is truly indicative of publication bias. An inventory of randomized controlled vitamin D trials registered in the NIH clinical trial registry (www.clinicaltrials.gov) did not provide any evidence of unpublished results from pre-registered trials, indicating that publication bias may not be a major problem in this field of research.

The relatively large treatment effect (OR 0.64) in combination with high absolute risks of RTI in placebo-treated subjects resulted in low NNTs ranging from 9 to 33. These results indicate that a limited number of individuals would require three months treatment with vitamin D in order to avoid an episode of RTI. Considering the therapy's low cost and general safety, this suggests a reasonable cost-effectiveness. However, a complete pharmacoeconomic evaluation is beyond the scope of this study and would require precise estimates of RTI incidence, treatment costs and the costs associated with RTI.

### Limitations

The results of this analysis should be interpreted with caution, due to a number of important limitations. Firstly, there was a large heterogeneity among studies. Hence, the pooled estimate may be of limited guidance when predicting the efficacy of vitamin D in individual patients, since the estimate reflects the average effect in a number of subpopulations. In the meta-regressions, only administration interval was identified as a potential source of such heterogeneity. Previous individual-level analyses have identified baseline 25(OH)D levels as a predictor of outcome [26] and the lack of such an effect in our trial may reflect a lack of power in trial-level analyses. Secondly, potential publication bias is a factor that should be taken very seriously, since it could easily exaggerate the effect or even simulate therapeutic effect when none exists. The shape of the funnel plot was such that an element of publication bias could not be ruled out, even though the formal test for funnel plot asymmetry was non-significant. Seemingly, the asymmetry could largely be explained by differences in administration interval of vitamin D and pre-registrations of RCTs did not indicate selective publishing, but the potential influence of publication bias should nevertheless be kept in mind when interpreting the results. Thirdly, within-trial bias could obviously have had an effect on the results. Fortunately, only two studies were identified as being at high risk of bias, and exclusion of these studies had only modest influence on the outcome (OR 0.67 vs OR 0.64 in all 11 studies). However, even studies with acceptably low risk of bias differ with regard to the absolute risk, and a pooled estimate from several studies of different quality is more likely to be influenced by bias compared to a single large trial of very high quality.

## Conclusions

Aggregated evidence from 11 randomized controlled trials indicates that supplementation with vitamin D could be an effective means of preventing respiratory tract infection. However, due to heterogeneity of included studies and possible publication bias in the field, these results should be interpreted with caution. Thus, additional studies addressing the impact of dosing regimen and choice of target population are warranted before definite conclusions can be drawn.

## Supporting Information

Checklist S1 **PRISMA checklist for reporting of systematic reviews and meta-analyses.**
(DOC)

Figure S1 **Influence analysis.** Error bars indicate 95% confidence intervals of summary effect estimates after exclusion of a single study.
(TIF)

Figure S2 **Funnel plot with pseudo 95% confidence limits.** Filled circles represent studies with low risk of within-study bias, open circles denote studies with high risk.
(TIF)

Protocol S1 **Pre-registered study protocol.** Meta-analysis study protocol pre-registered at the PROSPERO international register of systematic reviews (registration number CRD42013003530).
(PDF)

Table S1 **Data items extracted from eligible studies.**
(DOCX)

Table S2 **Risk of bias in included studies, as assessed with the Cochrane Collaboration's tool for assessing risk of bias in randomized trials** [**13**] **.**
(DOCX)

Table S3 **Adverse events in the included studies.** AE, adverse event; SAE, severe adverse event. AE- and SAE-column: total number of events; vitamin D-group and Placebo-group. #Refers to the original study by Aloia et al, Arch of Intern Med, 2005.
(DOCX)
