# Supplementary figures and images for: Vitamin D and Respiratory Tract Infections: A Systematic Review and Meta-Analysis of Randomized Controlled Trials
Source: PLoS One. 2013 Jun 19;8(6):e65835. doi: 10.1371/journal.pone.0065835 (PMC3686844; doi:10.1371/journal.pone.0065835)

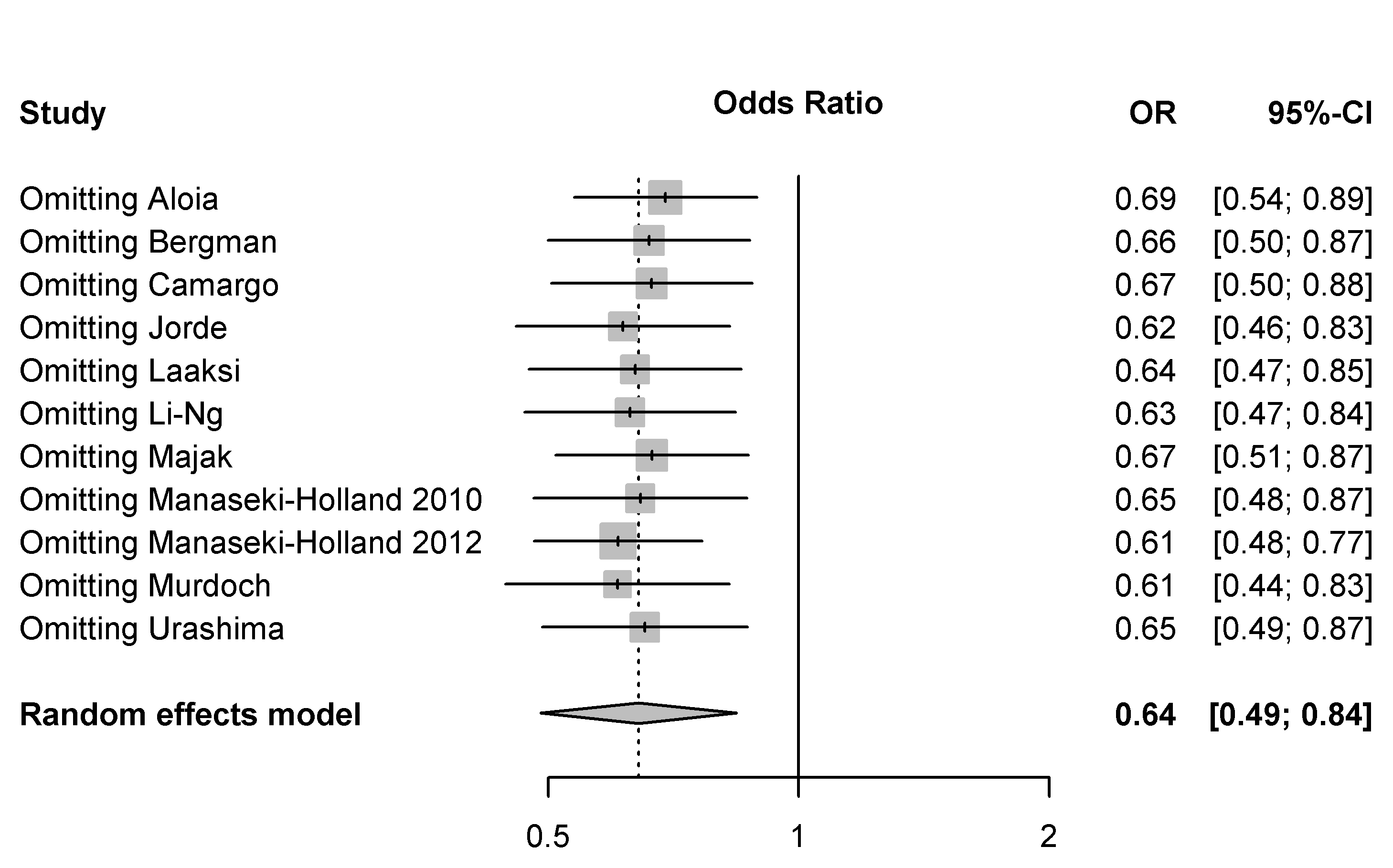

Supplement: Figure S1 — Influence analysis. Error bars indicate 95% confidence intervals of summary effect estimates after exclusion of a single study. (TIF) [file pone.0065835.s002.tif]
