# Supplementary material for: Vitamin D and Respiratory Tract Infections: A Systematic Review and Meta-Analysis of Randomized Controlled Trials
Source: PLoS One. 2013 Jun 19;8(6):e65835. doi: 10.1371/journal.pone.0065835 (PMC3686844; doi:10.1371/journal.pone.0065835)
Supplement: Protocol S1 — Pre-registered study protocol. Meta-analysis study protocol pre-registered at the PROSPERO international register of systematic reviews (registration number CRD42013003530). (PDF) [file pone.0065835.s004.pdf]

Review title and timescale

1 **Review title**

Give the working title of the review. This must be in English. Ideally it should state succinctly the interventions or exposures being reviewed and the associated health or social problem being addressed in the review.

Vitamin D and respiratory tract infection: a systematic review and meta-analysis of randomized controlled trials

2 **Original language title**

For reviews in languages other than English, this field should be used to enter the title in the language of the review. This will be displayed together with the English language title.

3 **Anticipated or actual start date**

Give the date when the systematic review commenced, or is expected to commence.

17/12/2012

4 **Anticipated completion date**

Give the date by which the review is expected to be completed.

06/01/2013

5 **Stage of review at time of this submission**

Indicate the stage of progress of the review by ticking the relevant boxes. Reviews that have progressed beyond the point of completing data extraction at the time of initial registration are not eligible for inclusion in PROSPERO. This field should be updated when any amendments are made to a published record.

Review stage	Started	Completed
Preliminary searches	No	Yes
Piloting of the study selection process	No	Yes
Formal screening of search results against eligibility criteria	No	Yes
Data extraction	No	Yes
Risk of bias (quality) assessment	No	Yes
Data analysis	No	Yes
Prospective meta-analysis	No	Yes

Provide any other relevant information about the stage of the review here.

Review team details

6 **Named contact**

The named contact acts as the guarantor for the accuracy of the information presented in the register record.

Jonatan Lindh

7 **Named contact email**

Enter the electronic mail address of the named contact.

jonatan.lindh@ki.se

8 **Named contact address**

Enter the full postal address for the named contact.

Dept. of Clinical Pharmacology, C1-68 14186 Stockholm Sweden

9 **Named contact phone number**

Enter the telephone number for the named contact, including international dialing code.

+46858581201

10 **Organisational affiliation of the review**

Full title of the organisational affiliations for this review, and website address if available. This field may be completed as 'None' if the review is not affiliated to any organisation.

None

Website address:

11 **Review team members and their organisational affiliations**

Give the title, first name and last name of all members of the team working directly on the review. Give the organisational affiliations of each member of the review team.

Title	First name	Last name	Affiliation
Dr	Jonatan	Lindh	Dept. of Laboratory Medicine, division of Clinical Pharmacology, Karolinska Insitutet
Dr	Peter	Bergman	Dept. of Laboratory Medicine, division of Clinical Microbiology, Karolinska Insitutet
Dr	Linda	Björkhem-Bergman	Dept. of Laboratory Medicine, division of Clinical Pharmacology, Karolinska Insitutet
Dr	Åsa	Lindh	Dept. of Psychiatry, St Göran's Hospital, Stockholm

12 **Funding sources/sponsors**

Give details of the individuals, organizations, groups or other legal entities who take responsibility for initiating, managing, sponsoring and/or financing the review. Any unique identification numbers assigned to the review by the individuals or bodies listed should be included.

Fundings from Karolinska Institutet and Karolinska University Hospital

13 **Conflicts of interest**

List any conditions that could lead to actual or perceived undue influence on judgements concerning the main topic investigated in the review.

Are there any actual or potential conflicts of interest?

None known

14 **Collaborators**

Give the name, affiliation and role of any individuals or organisations who are working on the review but who are not listed as review team members.

Title	First name	Last name	Organisation details
Ms	Marine	Andersson	Dept. of Laboratory Medicine, division of Clinical Pharmacology, Karolinska Insitutet

Review methods

15 **Review question(s)**

State the question(s) to be addressed / review objectives. Please complete a separate box for each question.

Is substitution with vitamin D an effective means of preventing respiratory tract infections?

16 **Searches**

Give details of the sources to be searched, and any restrictions (e.g. language or publication period). The full search strategy is not required, but may be supplied as a link or attachment.

MEDLINE: MeSH-indexed publications were searched with the following query: Vitamin D"[Mesh] AND ("Respiratory Tract Infections"[Mesh] OR "Infection"[Mesh]). For publications which had not yet been subjected to MeSH-indexing the following query was used: "vitamin D" AND infection AND (publisher[sb] OR in process[sb]). In addition, congress abstracts and review article reference lists will be used to identify eligible trials.

17 **URL to search strategy**

If you have one, give the link to your search strategy here. Alternatively you can e-mail this to PROSPERO and we will store and link to it.

18 **Condition or domain being studied**

Give a short description of the disease, condition or healthcare domain being studied. This could include health and wellbeing outcomes.

Respiratory tract infections (viral or bacterial). Tuberculosis is excluded.

19 **Participants/population**

Give summary criteria for the participants or populations being studied by the review. The preferred format includes details of both inclusion and exclusion criteria.

Individuals (healthy or patients) prospectively treated with vitamin D in a randomized, placebocontrrolled setting.

20 **Intervention(s), exposure(s)**

Give full and clear descriptions of the nature of the interventions or the exposures to be reviewed

Vitamin D substitution, without restrictions regarding dose, route of administration or dosage interval.

21 **Comparator(s)/control**

Where relevant, give details of the alternatives against which the main subject/topic of the review will be compared (e.g. another intervention or a non-exposed control group).

Placebo.

22 **Types of study to be included initially**

Give details of the study designs to be included in the review. If there are no restrictions on the types of study design eligible for inclusion, this should be stated.

Randomized controlled trials.

23 **Context**

Give summary details of the setting and other relevant characteristics which help define the inclusion or exclusion criteria.

24 **Primary outcome(s)**

Give the most important outcomes.

Respiratory tract infection (RTI), as defined in the individual trials. If available, proportion of patient experiencing one or more RTIs will be used. If unavailable, other endpoints reflecting incident RTI (e.g. number of RTI:s) will be used.

Give information on timing and effect measures, as appropriate.

25 **Secondary outcomes**

List any additional outcomes that will be addressed. If there are no secondary outcomes enter None.

None planned.

Give information on timing and effect measures, as appropriate.

26 **Data extraction, (selection and coding)**

Give the procedure for selecting studies for the review and extracting data, including the number of researchers involved and how discrepancies will be resolved. List the data to be extracted.

Identified titles and abstracts will be searched and potentially eligible studies will be retrieved in full text. Full text articles will be extracted independently by two researchers, and discrepancies will be reolved by consensus. Data to be extracted include: Publication details (year, authors etc.), patient characteristics (age, gender distribution, number of subjects, inclusion and exclusion criteria, vitamin D levels), treatment (vitamin D dose, dosage interval, route of administration), RTI definition, RTI being primary or secondary endpoint. All outcome measures including point estimates, SDs, SEs, confidence intervals, confounding adjustments etc.

27 **Risk of bias (quality) assessment**

State whether and how risk of bias will be assessed, how the quality of individual studies will be assessed, and whether and how this will influence the planned synthesis.

Quality assessment by means of Cochrane Collaboration's tool for assessing risk of bias in randomised controlled trials. Funnel plots and Begg-Mazumdar's test for funnel plot asymmetries.

28 **Strategy for data synthesis**

Give the planned general approach to be used, for example whether the data to be used will be aggregate or at the level of individual participants, and whether a quantitative or narrative (descriptive) synthesis is planned. Where appropriate a brief outline of analytic approach should be given.

If possible, continuous outcomes will be converted to odds ratios for pooling with odds ratios from studies with binary outcomes. Log-transformed odds ratios will be poooled in a random effects meta-analysis .

29 **Analysis of subgroups or subsets**

Give any planned exploration of subgroups or subsets within the review. 'None planned' is a valid response if no subgroup analyses are planned.

Subgroups that will be explored include patients vs healthy individuals, studies reporting RTI as a primary vs secondary outcome, studies giving vitamin D as daily doses vs infrequent bolus doses. In addition, meta-regressions will be used to investigate the influence of baseline vitamin D levels, vitamin D dose, treatment duration, and age.

Review general information

30 **Type of review**

Select the type of review from the drop down list.

Treatment

31 **Language**

Select the language(s) in which the review is being written and will be made available, from the drop down list. Use the control key to select more than one language.

English

Will a summary/abstract be made available in English?

Yes

32 **Country**

Select the country in which the review is being carried out from the drop down list. For multi-national collaborations select all the countries involved. Use the control key to select more than one country.

Sweden

33 **Other registration details**

List places where the systematic review title or protocol is registered (such as with The Campbell Collaboration, or The Joanna Briggs Institute). The name of the organisation and any unique identification number assigned to the review by that organization should be included.

34 **Reference and/or URL for published protocol**

Give the citation for the published protocol, if there is one.

Give the link to the published protocol, if there is one. This may be to an external site or to a protocol deposited with CRD in pdf format.

35 **Dissemination plans**

Give brief details of plans for communicating essential messages from the review to the appropriate audiences.

Results will be published in an international, peer-reviewed journal.

Do you intend to publish the review on completion?

Yes

36 **Keywords**

Give words or phrases that best describe the review. (One word per box, create a new box for each term)

Vitamin D

Respiratory tract infection

Infection

37 **Details of any existing review of the same topic by the same authors**

Give details of earlier versions of the systematic review if an update of an existing review is being registered, including full bibliographic reference if possible.

38 **Current review status**

Review status should be updated when the review is completed and when it is published.

Completed but not published

39 **Any additional information**

Provide any further information the review team consider relevant to the registration of the review.

40 **Details of final report/publication(s)**

This field should be left empty until details of the completed review are available.
Give the full citation for the final report or publication of the systematic review.

Give the URL where available.
