# Supplementary material for: Vitamin D and Respiratory Tract Infections: A Systematic Review and Meta-Analysis of Randomized Controlled Trials
Source: PLoS One. 2013 Jun 19;8(6):e65835. doi: 10.1371/journal.pone.0065835 (PMC3686844; doi:10.1371/journal.pone.0065835)
Supplement: Table S1 — Data items extracted from eligible studies. (DOCX) [file pone.0065835.s005.docx]

| PubMed unique identifier (PMID) |
| --- |
| Full PubMed reference, including authors, title, journal, volume, pages, and year of publication. |
| Country where study was performed |
| Latitude of study site |
| Vitamin D dose and dosage interval |
| Vitamin D route of administration |
| Vitamin D average daily dose |
| Follow-up time |
| Number in included individual (per study group and total) |
| Average age of participants |
| Gender distribution of participants |
| Vitamin D concentrations in plasma (at baseline and follow-up, per study group) |
| Definition of infection |
| Inclusion criteria |
| Exclusion criteria |
| RTI as primary endpoint (yes/no) |
| Healthy subjects (yes/no) |
| RTI-associated outcome measure(s) (incl. e.g. mean, SD, OR, RR, confidence intervals, no. of participants) |
| Outcome measures other than RTI |
| Additional covariates recorded |
