# Supplementary material for: Vitamin D and Respiratory Tract Infections: A Systematic Review and Meta-Analysis of Randomized Controlled Trials
Source: PLoS One. 2013 Jun 19;8(6):e65835. doi: 10.1371/journal.pone.0065835 (PMC3686844; doi:10.1371/journal.pone.0065835)
Supplement: Table S2 — Risk of bias in included studies, as assessed with the Cochrane Collaboration's tool for assessing risk of bias in randomized trials [13] . (DOCX) [file pone.0065835.s006.docx]

| **Study** |  | **Random sequence generation** | **Allocation concealment** | **Blinding of participants and personnel** | **Blinding of outcome assessment** | **Incomplete outcome data** | **Selective reporting** | **Other bias** |
| --- | --- | --- | --- | --- | --- | --- | --- | --- |
| Camargo[^21^](#_ENREF_20) | 2012 | Low | Low | Unclear | Unclear | Low | Low | Low |
| Laaksi[^25^](#_ENREF_24) | 2010 | Low | Low | Low | Low | Unclear | Low | Low |
| Aloia[^17^](#_ENREF_16) | 2007 | Low | Low | Low | Low | Unclear | High | Low |
| Urashima[^32^](#_ENREF_31) | 2010 | Low | Low | Low | Low | Unclear | Low | Low |
| Lehouck[^26^](#_ENREF_25) | 2012 | Low | Low | Low | Low | Low | Low | Low |
| Manaseki[^30^](#_ENREF_29) | 2010 | Low | Low | Low | Low | Low | Low | Low |
| Manaseki[^29^](#_ENREF_28) | 2012 | Low | Low | Low | Low | Low | Low | Low |
| Murdoch[^31^](#_ENREF_30) | 2012 | Low | Low | Low | Low | Low | Low | Low |
| Li-Ng[^27^](#_ENREF_26) | 2009 | Low | Low | Low | Low | Unclear | Low | Low |
| Bergman[^19^](#_ENREF_18) | 2012 | Low | Low | Low | Low | Low | Low | Low |
| Majak[^28^](#_ENREF_27) | 2011 | Low | Low | Low | Low | Low | Low | Low |
| Jorde[^23^](#_ENREF_22) | 2012 | Unclear | Low | Low | Low | High | Low | Unclear |
