# Supplementary material for: Vitamin D and Respiratory Tract Infections: A Systematic Review and Meta-Analysis of Randomized Controlled Trials
Source: PLoS One. 2013 Jun 19;8(6):e65835. doi: 10.1371/journal.pone.0065835 (PMC3686844; doi:10.1371/journal.pone.0065835)
Supplement: Table S3 — Adverse events in the included studies. AE, adverse event; SAE, severe adverse event. AE- and SAE-column: total number of events; vitamin D-group and Placebo-group. #Refers to the original study by Aloia et al, Arch of Intern Med, 2005. (DOCX) [file pone.0065835.s007.docx]

| ADVERSE EVENTS | YEAR | AE | SAE | Comments |
| --- | --- | --- | --- | --- |
| Aloia[17]# | 2007 | 222 | 15; D:8, P:7 | No events were related to study drug |
| Bergman[19] | 2012 | 94; D: 38, P: 56 | 2; D:1, P: 1 | No events were related to study drug |
| Camargo[21] | 2012 |  |  | No significant adverse events detected |
| Jorde[23] | 2012 |  |  | Not mentioned in the text |
| Laaksi[25] | 2010 | 3; D:2, P:1 |  |  |
| Lehouck[26] | 2012 |  |  | D: 4 cases of mild hypercalcemia |
| Li-Ng[27] | 2009 | 72; D:38, P:34 | 5; D:3, P:2 |  |
| Majak[28] | 2011 |  |  | Not mentioned in the text |
| Manaseki-Holland[30] | 2010 |  |  | No significant adverse events detected |
| Manaseki-Holland[29] | 2012 |  |  | No significant adverse events detected |
| Murdoch[31] | 2012 | 1492; D:700, P:792 | 40; D:21, P:19 | No events were related to study drug |
| Urashima[32] | 2010 |  |  | No significant adverse events detected |
